# Focal cortical dysplasias in autism spectrum disorders

**DOI:** 10.1186/2051-5960-1-67

**Published:** 2013-10-11

**Authors:** Manuel F Casanova, Ayman S El-Baz, Shweta S Kamat, Brynn A Dombroski, Fahmi Khalifa, Ahmed Elnakib, Ahmed Soliman, Anita Allison-McNutt, Andrew E Switala

**Affiliations:** 1 Department of Psychiatry & Behavioral Sciences, University of Louisville, Louisville, KY 40202, USA; 2 Department of Bio-engineering, University of Louisville, Louisville, KY 40292, USA; 3 Department of Anatomical Sciences & Neurobiology, University of Louisville, LearningRx Louisville-Springhurst, Louisville, KY 40202, USA; 4 School of Medicine, University of Louisville, Louisville, KY 40202, USA

**Keywords:** Autistic spectrum disorder, Frontal lobe, Malformations of cortical development, Cortical width

## Abstract

**Background:**

Previous reports indicate the presence of histological abnormalities in the brains of individuals with autism spectrum disorders (ASD) suggestive of a dysplastic process. In this study we identified areas of abnormal cortical thinning within the cerebral cortex of ASD individuals and examined the same for neuronal morphometric abnormalities by using computerized image analysis.

**Results:**

The study analyzed celloidin-embedded and Nissl-stained serial full coronal brain sections of 7 autistic (ADI-R diagnosed) and 7 age/sex-matched neurotypicals. Sections were scanned and manually segmented before implementing an algorithm using Laplace’s equation to measure cortical width. Identified areas were then subjected to analysis for neuronal morphometry. Results of our study indicate the presence within our ASD population of circumscribed foci of diminished cortical width that varied among affected individuals both in terms of location and overall size with the frontal lobes being particularly involved. Spatial statistic indicated a reduction in size of neurons within affected areas. Granulometry confirmed the presence of smaller pyramidal cells and suggested a concomitant reduction in the total number of interneurons.

**Conclusions:**

The neuropathology is consistent with a diagnosis of focal cortical dysplasia (FCD). Results from the medical literature (e.g., heterotopias) and our own study suggest that the genesis of this cortical malformation seemingly resides in the heterochronic divisions of periventricular germinal cells. The end result is that during corticogenesis radially migrating neuroblasts (future pyramidal cells) are desynchronized in their development from those that follow a tangential route (interneurons). The possible presence of a pathological mechanism in common among different conditions expressing an autism-like phenotype argue in favor of considering ASD a “sequence” rather than a syndrome. Focal cortical dysplasias in ASD may serve to explain the high prevalence of seizures and sensory abnormalities in this patient population.

## Background

Neurologists habitually regard autism as a disease affecting the cerebral cortex [1]. The presence of seizures, abnormalities of higher cognitive functions, and absence of either spasticity or vision loss, supports this contention. It is therefore unsurprising that neuropathological findings of a dysplastic nature have been described in the cerebral cortex of autistic individuals. The brunt of dysplastic changes appears to be within the neocortex wherein abnormalities of gyrification and laminar architecture are often found. These findings include effacement of the normal lamination pattern, minicolumnar abnormalities, and variations in neuronal density [1-4]. In addition to the previously mentioned microscopic changes, at gross examination, brains of autistic individuals have shown malformations in the form of hyperconvolution [5], misdirected gyri [6], and polymicrogyria [7].

Among the earliest reports of cortical abnormalities in autism was the claim made by Bauman and Kemper of indistinct lamination of the anterior cingulate gyrus in 5 out of their 6 cases [8]. However, it was Bailey and colleagues who first emphasized the significance of abnormalities of the cerebral cortex in autism [5]. In their series, 4 out of 6 patients had both abnormal neuronal densities and lamination of the frontal cortex, and 1 patient showed an increased number of neurons within the molecular layer. Several years later, Mukaetova-Landinska and colleagues examined sections of MAP2 immunocytochemistry from BA9 and BA10 for dendritic complexity [9]. The results of the study showed ill-defined cortical layers and a reduced level of MAP2 expression in the neuronal soma and dendrites of autistic individuals. More recently, Hutsler and colleagues used thickness and lamination as proxy measurements for cortical organization when studying histological sections of eulaminate cortex (BA7, BA9, and BA21) in 8 autistic spectrum disorder individuals and an equal number of controls [10]. Between-group differences in cortical thickness were judged non-significant. Qualitative examination revealed cell clustering and an increased number of cells in lamina I and in the subplate region. A later study using the same patient population evaluated the transition zone between the cortical gray and white matter by overlaying a sigmoid function in binary images [11]. Their results indicated an indistinct boundary accounted, possibly, by the presence of supernumerary neurons beneath the cortical plate. The authors speculated that the presence of supernumerary neurons was the result of either a migrational defect or failed apoptosis within the subplate region [12]. Studies on whole brain serial sections by Wegiel and colleagues suggest that defects of neurogenesis and neuronal migration account for described dysplastic changes [13].

In this study we examined for dysplastic abnormalities in postmortem brains of autistic individuals by measuring the cortical thickness of full hemispheric coronal sections. We decided on pursuing a postmortem study, rather than neuroimaging, in order to optimize the resolution of the gray-white matter transition zone. Previous studies suggest that using neuroimaging to measure cortical thickness may provide for inaccurate results due to blurring of the transition zone by an increased number of neurons within the subcortical white matter [10,11,14]. Embedding in celloidin was selected so as to minimize tissue shrinkage. Artifacts accrued to handling large sections for mounting on slides were avoided, in part, by manually segmenting the sections.

## Methods

### Clinical dataset

The diagnosis for each autistic patient was established postmortem by the Autism Tissue Program (ATP). A certified rater and trainer arranged for a postmortem visit with the family to obtain, with written consent, medical and clinical information via a questionnaire that included the Autism Diagnostic Interview-Revised (ADI-R).

The Harvard Brain Tissue Resource Center (HBTRC) questionnaire was modified to include autism-specific questions for ATP use. The information obtained included: donor and respondent identifying information; ethnicity, handedness and known exposure to hazardous materials; diagnostic information including dates and physician; genetic tests; pre- and postnatal medical history; immunization, medication, and hospitalization information; family history and additional information about donor participation in any training or research studies such as imaging, medication trials, and/or genetic studies. The supporting documents such as autopsy reports, death certificates, medical, clinical, and/or educational records were obtained at the time of the home visit or by sending written requests, signed by the legal next-of-kin, to named providers.

Whole, formalin fixed, cerebral hemispheres from the ATP had already been processed at the Institute for Basic Research in Developmental Disabilities, Staten Island, New York. Seven were obtained from individuals diagnosed with autism spectrum disorder, and seven donations from neurotypical individuals were selected to match the ASD cases by age and sex where possible (Table 1). Tissue preparation was according to the method of Heinsen and Heinsen [15]. Serial, coronal sections 200 μm thick, spaced 1.2 mm apart and spanning the entire hemisphere, were stained with gallocyanine and mounted on glass slides. These slides were later shipped to the authors at the University of Louisville for the present work.

**Table 1:** ATP donors with autism spectrum disorder and age-matched controls used for this experiment

	**ATP case #**	**Age**	**Sex**	**Seizures**	**Medication history**	**Cause of death**
	**Autism spectrum disorder**					
1.	UMB-1627	5.0	F	N	no medication	Multiple injuries/Motor vehicle accident
2.	AN13961	7.5	M	Y	Phenobarbital	Drowning
					Tegretol	
					Albuterol	
3.	AN00754	13.–	M	Y	Trileptol	Epilepsy
					Trazadone	
4.	IBR-425-02	4.2	M	N	Asthma medication NOS	Drowning, Hypothermia
5.	AN19511	8.–	M	N	Peridex	Rhabdomyosarcoma
					Nystatin	
					G-CSF	
					Benadryl	
					Phenergan	
					Dexamethasone	
					Morphine	
6.	AN02338	17.2	F	N	Paxil	Cardiac arrest/Dilated cardiomyopathy
7.	AN09730	22.9	M	Y	Neurontin	Choking/Epilepsy
					Thioridazine	
					Zonegran	
					Fish oil	
					Flaxseed oil	
					CoEnzyme Q10	
	**Neurotypical**					
1.	UMB-1499	4.5	F	N	N/A	Myocarditis
2.	UMB-4898	7.7	M	N	N/A	Drowning
3.	BTB-3638	14.3	M	N	N/A	Electrocution
4.	AN02456	4.–	F	N	N/A	Bronchopneumonia / Post-surgical procedure
5.	UMB-1708	8.1	F	N	N/A	Multiple injuries
6.	UMB-1843	15.9	F	N	N/A	Multiple injuries / Motor vehicle accident
7.	UMB-1646	23.2	M	N	N/A	Rupture of spleen

### Image processing

Imaging was done at 800 dpi (1 pixel = 317.5 μm) using a ScanMaker i900 flatbed scanner for transparent media (Microtek, Santa Fe Springs, California). The outer and inner cortical boundaries were manually traced. Five slides, randomly selected, were traced by three different individuals in order to determine inter-rater reliability.

**Figure 1:**
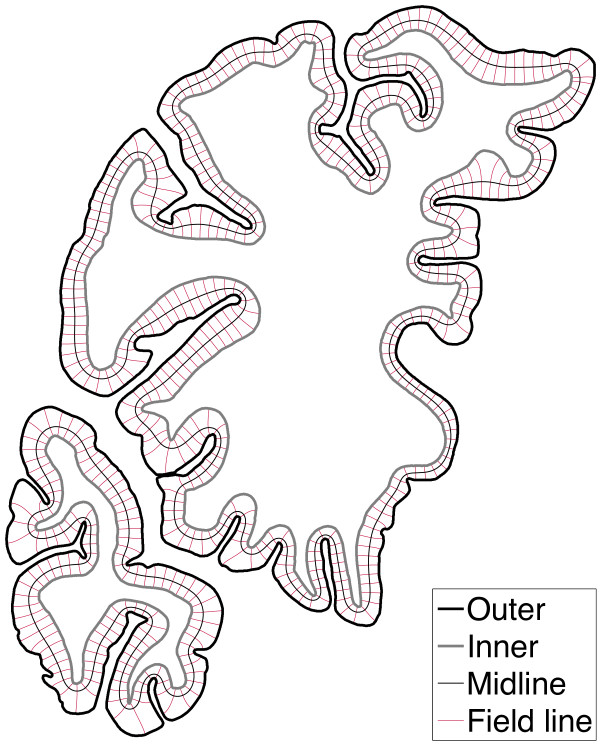
**Solution of the Laplace equation inside the cortical ribbon.** The outer contour is the lamina I/lamina II boundary *φ* = –1 ; the inner contour is the white matter boundary *φ* = +1; and the midline is the equipotential *φ* = 0. Field lines are displayed spaced at 1 mm intervals along the midline.

Cortical thickness was estimated using an algorithm based on electrostatics [16]. The inner and outer surfaces were treated as equipotential surfaces at +1 V and -1 V, respectively. The Laplace equation was solved to find the potential *φ* within the grey matter between these two boundaries. The thickness *t* of the cortex at a point is then given by the length of the field line through that point, where field lines are defined as curves extending from the inner surface to the outer, everywhere parallel to ∇*φ*. Thus is *t* unambiguously defined everywhere inside the grey matter, since field lines do not cross one another. Once computed, *t* was sampled on the medial surface, i.e. the equipotential surface at *φ* = 0. The local curvature *κ* of the brain surface was calculated on the same equipotential (Figure 1).

Identification of possible dysplastic regions began with matching individual slides between ASD-neurotypical pairs according to stereotaxic coordinate *y*. Coronal slices were positioned in MNI152 space so that *y* = -106 mm at the occipital pole, *y* = 73 mm corresponds with the frontal pole, and the slide containing the anterior commissure has position *y* = 0. The sampling distribution of *t* in each slide from the neurotypical case was used to estimate “fences” beyond which statistical outliers would fall, using the method of Hubert and Van der Veeken [17]. Regions of the corresponding slide in the matched ASD case where *t* fell below the lower fence were identified as possible dysplasias.

We undertook detailed preliminary study of the purported dysplasia in twelve identified regions from two ASD donor’s brains. These were contrasted with twelve homologous regions from their respective control donors. High-resolution images (1 pixel = 0.74 μm) were obtained within these regions of interest, spanning the full width of the cortex. Segmentation of pyramidal cells followed a three step process. First, we employed a pixel-wise relaxation based on a maximum a posteriori (MAP) estimate of a pairwise energy function of a generalized, two-dimensional Gauss-Markov random field (GGMRF) probabilistic model [18,19] in order to reduce the effect of image noise. Secondly, we used a marker-based watershed approach [20] to get an initial segmentation of the neurons from the MGRF-relaxed image, obtained in the first step. Finally, for each initially segmented neuron, we refined its segmentation based on using a local iterative thresholding approach. The optimum threshold is obtained in a way that maximizes the distance between the average signal intensities of the initially segmented neuron region and its background. Segmented binary images were analyzed using Boolean spatial statistical model [21] and also granulometry.

## Results

Age at death did not differ by more than 1.3 years between paired individuals (Table 1). Individuals were less well matched for sex, with two male ASD donors being paired with female neurotypical controls. Three of the individuals with autism had suffered from seizures. Two ASD donors, and the matched case of one of them, had died from drowning.

Inter-rater reliability of the manual segmentation of the cerebral cortex was *AC*_1_ = 87%, using Gwet’s agreement coefficient [22].

**Figure 2:**
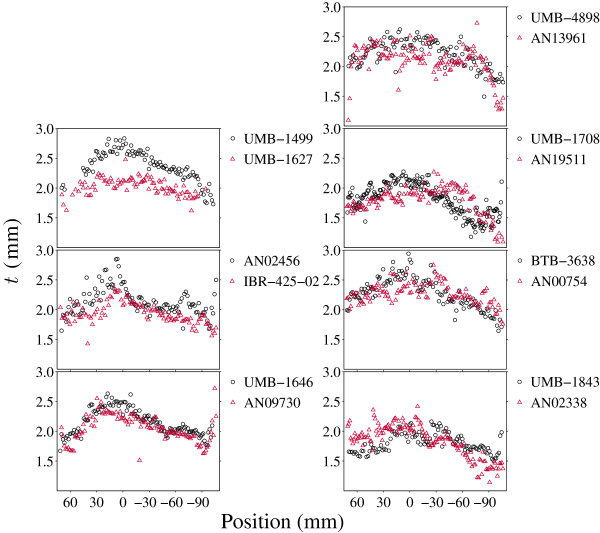
**Mean *t *in each brain section.** The seven pairs are plotted individually, with measurements from the ASD donor indicated by triangles, and circles for the matched neurotypical donor. Note that the horizontal axis scale is not uniform. Average thickness for each slide is plotted with respect to the slide’s corresponding position in stereotaxic space. Six out of seven ASD patients showed diminutions within prefrontal cortex as defined by the AC.

Considering *t* as a function of *y* alone, mean cortical thickness was reduced in ASD in the vicinity of, and somewhat anterior to, the anterior commissure (AC) (Figure 2). Six out of seven ASD patients showed diminutions within prefrontal cortex as defined by the AC. The anterior commissure was taken as an anatomical landmark for the prefrontal lobe. Use of the AC as a defining criterion omits the motor cortex from consideration but is a better established landmark than other points of demarcation [23].

**Figure 3:**
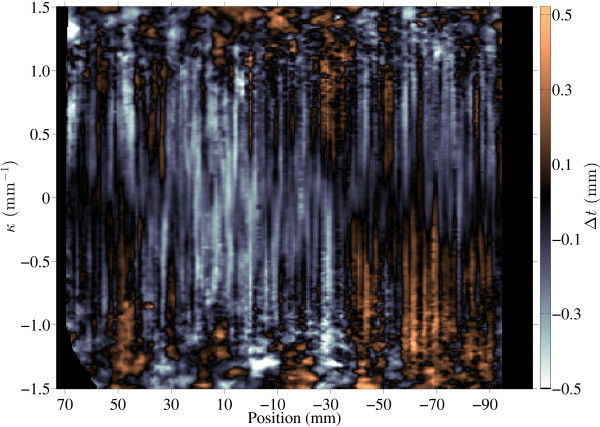
**Difference in *t *between the cerebral cortices of ASD and neurotypical donors with respect to position and curvature.** The horizontal axis is the same as in Figure 2 (73 mm = frontal pole; 0 = anterior commissure; -106 mm = occipital pole). Regions where the cortex is narrower in ASD are colored blue, while regions where the cortex is wider are colored orange. Data were averaged across all seven pairs.

Reduction in mean *t* is also seen near the occipital pole where findings were driven primarily by results in one patient (AN02338). When curvature is considered as well as position (Figure 3) reduction in thickness was furthermore seen in regions of positive curvature both anteriorly and posteriorly. Middle sections exhibited reductions in thickness equally distributed among the crest, sulci and flat face of gyri. Conversely, increased *t* was observed in posterior regions of negative curvature.

**Figure 4:**
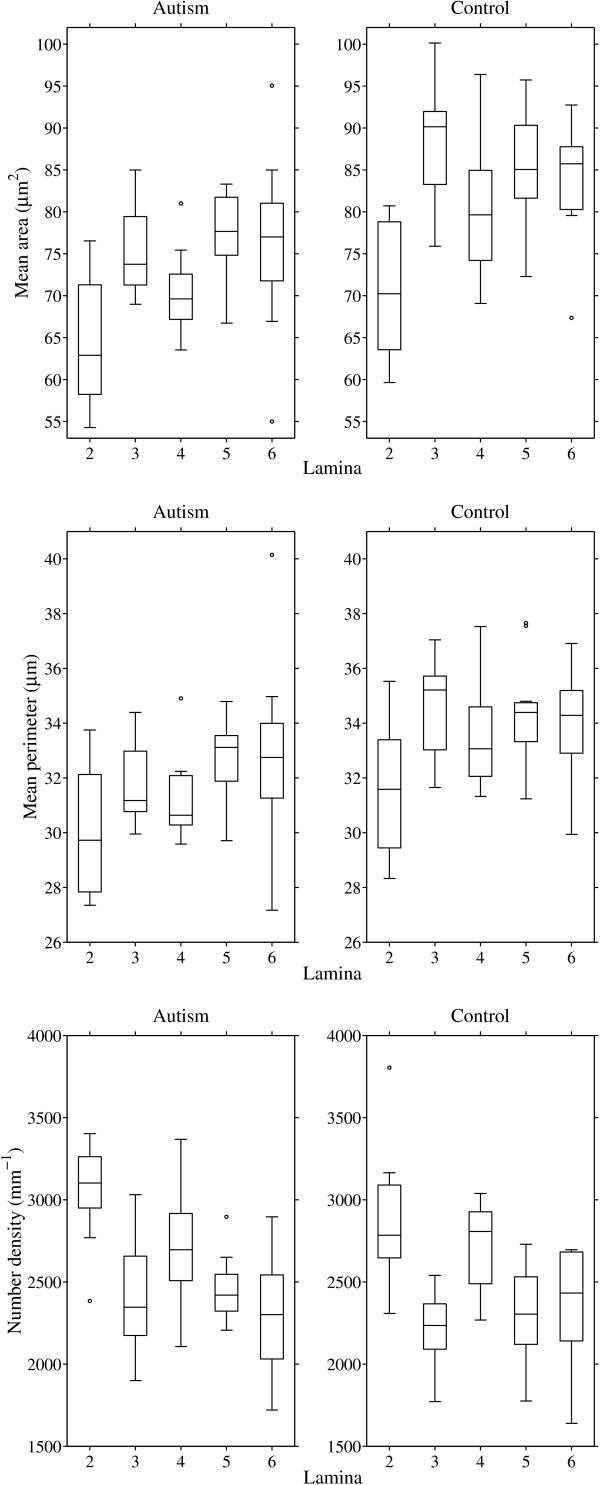
**Box plots of estimated Boolean model parameters by lamina.** The Boolean germ-grain model is completely characterized by three quantities: mean area and perimeter of the grain distribution, and intensity of the Poisson germ process.

**Figure 5:**
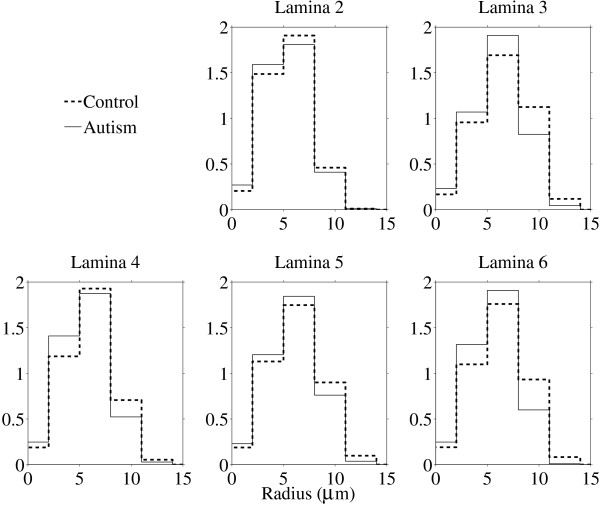
**Pattern spectrum by lamina, obtained by granulometry on segmented (binary) images.** Bar height (y axis) is proportional to the total area of stained objects with sizes falling within each bin. The spectra were averaged bin-wise over the twelve narrow cortical regions of interest from two ASD cases and the twelve corresponding regions from matched control cases.

Spatial statistics showed smaller average particle size (lower $\overset{¯}{A}$ and $\overset{¯}{U}$) and essentially the same particle density (*λ*) in ASD. Granulometry corroborated the finding with a downward shift in the mode of an apparently unimodal size distribution in ASD compared with controls. Findings were uniform across laminae II–VI (Figures 4 and 5).

The total number of individuals within our series and unavailability of some clinical data did not allow us to pursue a correlation between findings of cortical dysplasia and seizures. Cytoarchitectural characterization of dysplastic regions revealed absence of either dysmorphic or balloon neurons. There was no evidence of gliosis. The affected gyri were not mushroom shaped, nor did they acquire the shape of tubers. There was no evidence of ulegyria. Cellularity within the molecular layer was judged as normal. Some affected areas seemed dyslaminated but this was not evident in all cases.

## Discussion

**Figure 6:**
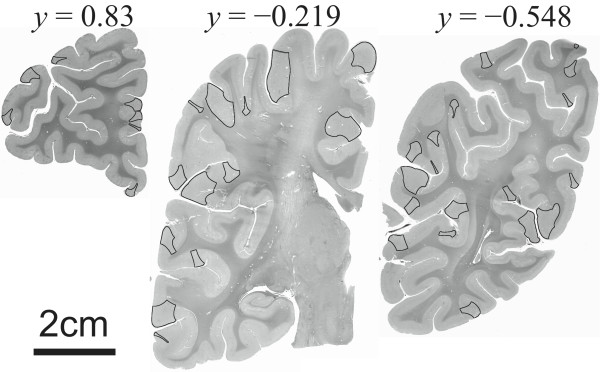
**Coronal sections through the brain of an autistic patient.** The overlay denotes those cortical areas showing abnormalities of cortical thickness. Towards both pole regions cortical thickness abnormalities preferentially affected the crest of gyri. Defects in cortical width tended to affect both crest and sulci of gyri in middle sections. Defects affecting crest of gyri were usually larger than those seen at the sulci of gyri. The *y*-coordinates are the normalized positions as in Figures 3 and 4.

Results of our study indicate the presence of multiple circumscribed dysplastic foci distributed throughout the cerebral cortex of autistic individuals. Described defects differed among affected individuals both in terms of location as well as overall size. Cortical abnormalities followed a rostro-caudal pattern being predominantly found at the crest of gyri near the pole regions and within the sulci, crest and flat face of gyri towards the middle sections of the brain (Figure 6). Regions occupied small portions of gyri that themselves seemed devoid of gross morphological abnormalities, e.g., tubers. Findings occurred in all ASD cases examined and involved all brain lobes but were most abundant within the prefrontal lobes.

The specific topography of dysplasias in ASD bears analogy to the rostrocaudal pattern of brain maturation associated with sulcation and gyrification [24]. These gradients are used to divide the cerebral cortex into functional domains [25]. Throughout evolution this patterning has remained intimately related to an increased number of stem cell divisions resulting in supernumerary ontogenetic columns or minicolumns [26]. Gradient-based-patterning mechanisms facilitate the preservation of the relative size and positions of functional parcellations despite increases in cortical surface area [25]. Disruption of this patterning mechanism (e.g. heterochronic division of periventricular germinal cells) may help explain the presence of both gyral and cerebral dominance abnormalities previously reported in the brains of autistic individuals [27,28] and the focal cortical dysplasias described in the present study.

In ASD, morphometry of neurons within affected areas revealed smaller neurons. Findings were present throughout layers II-VI of the cortex suggesting changes of anatomical elements in common throughout most of the cortical width. A histogram of sizes by granulometric analysis indicated a reduction of larger cells and an overall increase in size of the smaller ones. A possible interpretation to the reported findings is the diminution in size of larger pyramidal cells and a reduction in total numbers of smaller neuronal elements (interneurons). This supposition is strengthened by its ability to explain several findings from the literature:

1) In a previous study neuronal density in this patient population was found to be increased when thresholding by size in order to select for pyramidal cells [29]. Neuronal density in the present study, although normal, took into account a range in size inclusive of interneurons. When averaging the total number of both pyramidal cells and interneurons a reduction in the latter would tend to balance an increase in the former.

2) The total reduction of smaller neurons (interneurons) would be in keeping with reductions in the peripheral neuropil space of minicolumns reported in autism [30] and with symptoms suggestive of cortical hyperexcitability commonly reported in this patient population [31].

3) The size of a neuron’s cell body parallels the length of its axon. A long axon requires increased organelle machinery to sustain its higher metabolism. The presence of smaller pyramidal cells biases corticocortical connectivity towards shorter projections [29].

The dysplastic defects described in autism may be considered as exemplars of disturbed morphogenesis themselves stemming from an interference with corticogenesis. The dysplastic changes of the cerebral cortex coexist with heterotopias thus suggesting as a plausible pathogenic mechanism the heterochronic division of periventricular germinal cells and abnormalities of the ensuing migration of daughter cells to the cortex [13]. Disturbed minicolumnarity with defects within their peripheral neuropil space (i.e., the compartment holding most of the inhibitory cells) across the different cortical layers further suggests that once radially migrating neuroblasts reach the cortical plate they are desynchronized in their maturation from the tangentially migrating interneurons [4,30]. Evidence of periventricular germinal cell abnormalities is derived from neuropathological studies of idiopathic and syndromic autism. Abnormalities in the asymmetric division of periventricular germinal cells can account for the epigenetic heterotopias and minicolumnar abnormalities observed in idiopathic cases [13,29,32,33]. It is therefore not surprising that germinal matrix pathology is similarly evident in syndromic cases bearing an autism-like phenotype, such as congenital cytomegalovirus infection, antenatal cocaine exposure, extreme prematurity, tuberous sclerosis, and the Ehlers-Danlos syndrome [4].

### Focal cortical dysplasias

Malformations of the cortex are congenital morphological defects stemming from an intrinsically abnormal developmental process. Propitiating agents for malformations include medical illnesses, drugs, environmental agents, and genetic abnormalities. These abnormalities range in severity from minute defects invisible to the human eye to gross deficits involving large territories of the cerebral hemispheres (e.g., schizencephaly). Variability in severity depends on the timing, intensity, duration and nature of the inciting agent. Classification schemes for these malformations are based upon the model of cortical development proposed, to a large extent, by Sidman and Rakic [34]. This descriptive classification subtypes cortical malformations according to the timing of the defect during brain development. Defects are thus classified and ordered according to whether they involve aberrant cellular proliferation, neuronal migration, or cortical organization. As many cases fall outside the groupings of this classification, a fourth type comprised of miscellaneous entities is usually added [35]. In clinical practice, however, the different types of malformations often coexist in various combinations whose overlapping features make it difficult to tease each other apart [36].

Malformations are considered dysplastic if they evidence disorganized development. This definition differentiates dysplasias from other malformations due to incomplete morphogenesis, e.g., cortical malformations wherein the morphological features are characterized by underdevelopment due to a decrease in the number of cells (hypoplasia) and those where the tissue or organ fails to develop (aplasia) [37]. In the case of the cerebellum, for example, hypoplasia may be manifested as a smaller organ with fissures of normal size in comparison with the folia. Dysplasias of the cerebellum, on the other hand, display an abnormal pattern of folia and/or presence of heterotopic nodules [38].

Traditionally, focal cortical dysplasias were believed to be confined to one cerebral hemisphere or even to a specific brain region like the inferior frontal lobe [12,39]. More recently, the term has been expanded to include disparate entities varying in severity and histological findings [40,41]. Among the changes observed in FCD subtypes are variations in cortical thickness, compromised vertical organization with increased/persisting minicolumnar arrangements and aggregates of smaller neurons [42]. The findings are of interest as previous neuropathological studies in autism have similarly reported increased numbers of minicolumns with smaller neurons [29,32]. In essence, the present findings of smaller cells in thinned regions of the cerebral cortex and those of prior researchers (i.e., ectopic neurons, poorly defined gray-white matter boundary, increased neuronal density in layer I, and subcortical white matter, increased number of minicolumns) indicate the presence of focal dysplastic changes within the cerebral cortex of autistic individuals. These malformations describe disruptions of cellular proliferation, migration and cortical organization that start long before a person is born.

### Significance of findings

#### ***Frontal lobe symptoms***

In the present study a large number of dysplastic defects were observed in the frontal lobes of autistic individuals. This is not unexpected as the prefrontal lobe alone or its analogues account for approximately 29% of the total cortex of humans [43]. Besides the large area occupied by this brain region, the frontal lobes are characterized by their protracted development, a fact made most apparent by the late myelination of its axonal connections [44]. Modern neuroimaging studies suggest that the volume of the prefrontal white matter increases throughout childhood and continues beyond adolescence into young adulthood [45,46]. The protracted development of the frontal lobes makes it a better target for environmental exigencies to disturb its organization. Once affected, the role of the frontal lobe as a connectivity hub explains why damage to this area may result in widespread brain dysfunction. Abnormalities may result from both the failure to transfer incoming information and to the possibility of propagating potential errors.

The frontal lobe abnormalities described in this study help to explain why autism has been linked to a dysfunction of the executive system in managing multiple cognitive processes. In effect, one cognitive account of autism is the executive dysfunction theory that assumes how the presence of frontal lobe pathology leads to, among other symptoms, perseveration and an inability to shift attention [47]. The degree to which such deficits are associated to intellectual disability is unclear and remains the object of considerable discussion within the medical literature [48]. However, the absence of demonstrable executive deficits in a subgroup of autistic patients would argue against executive dysfunction being a core deficit of the condition [49]. The variable involvement of different areas of the brain, including the frontal lobes, by focal cortical dysplastic processes as shown in the present study helps explain the heterogeneity of clinical symptomatology in ASD.

#### ***Seizures***

Malformations of cortical development frequently result in varying combinations of intractable seizures, mental retardation, cerebral palsy, and focal neurological deficits [50]. Although microdysgenesis could be important in the pathophysiology of epilepsy, the basic mechanisms involved remain unknown [41]. Previous studies in autism have suggested a deficit in the inhibitory surround of minicolumns reflective of cellular networks prone to monotonically increasing avalanches of activity, i.e., a “rich-gets-richer” mechanism [30,51]. Furthermore, once an error enters such a system it will be propagated and amplified through downstream connections. It is thus unsurprising that cortical dysplasias are intrinsic epileptogenic lesions that commonly provide for intractable seizures [52] of multiple types [53] with remissions occurring in only a small proportion of patients [54]. Focal cortical dysplasia may thus serve as a causative factor or, in some instances, a propitiating factor for seizures. This cortical malformation may thus confer a susceptibility rendering the affected brain vulnerable to seizures after subsequent injuries such as febrile convulsions or head trauma [55,56].

#### ***Motor and sensory abnormalities***

Inhibition is crucial to the synchronization of neural networks wherein patterned connections give rise to organized behaviors. Specific cell assemblies that have interneurons (e.g., Wilson-Cowan Oscillator) are thought to participate as pattern generators within neural networks, e.g. spontaneous rhythmical activity patterns (as in the movement of tentacles) or as biological clocks [57]. These cell assemblies are poised within a hierarchical organization that includes central pattern generators, initiators, and controllers. Some of the functions sustained by these pattern generators include locomotor activities (e.g., posture, walking, reaching, grasping, manipulating) and fixed action patterns (i.e., stereotyped behaviors elicited by fixed stimuli). An inhibitory deficit could help explain abnormalities in locomotor skills, sleep disorders, extended periods of restlessness, and stereotypic movements observed in ASD [58,59].

Both pyramidal cells and interneurons display spontaneous activity so that synaptic inputs modulate their firing rate around a baseline rate. A lack or deficiency of inhibition (e.g., a diminished number of interneurons) may help depolarize existing neurons below the trigger point of their action potential. In this scenario otherwise weak signals add up thus providing spikes of activity. This phenomenon, called stochastic resonance, makes reference to the transmission of a weak signal in the presence of noise [60]. Stochastic resonance may provide an explanation to the high prevalence of sensory problems and their resultant behaviors that handicap many autistic patients.

## Conclusions

It is the opinion of the authors that the constellation of neuropathological abnormalities described in autism, along with its *formes fruste*, e.g., PDD-NOS (DSM-IV-TR), does not comprise a syndrome. The abnormalities in cortical width found in our study along with many previously described neuropathological findings in the literature, although heterogenous (i.e., varying from case to case), are in many cases linked through a cascading chain of events propitiated by disordered periventricular and rhombic lip germinal cell divisions. This is in contrast to a syndrome where the pathogenic relationships between manifested abnormalities are frequently not understood. The authors therefore propose that associated conditions expressing an autism-like phenotype may be related to a common risk factor.

In autism the presence of periventricular nodular and white matter heterotopias, and that of a minicolumnopathy, offer many pathophysiological commonalities [13,29,32]. The principle of parsimony suggests that neuropathological findings in autism are best seen as a pattern of morphologies stemming from a single original event: the heterochronic divisions of periventricular germinal cells. In this regard periventricular germinal cells offer a *locus minoris resistentiae* to the pathology of autism [4]. Findings are therefore more accurately described as a “sequence” or chain of variable initiating events all manifesting the clinical phenotype of autism.

In summary, this study describes the presence of dysplastic processes throughout the cerebral cortex of individuals with ASD as reflected in measurements of cortical thickness. Previous studies of indistinct cortical lamination and supernumerary neurons in both the molecular layer and white matter clearly indicate the presence of mild malformations stemming from a defect during corticogenesis. The occasional presence of subcortical heterotopias and nodular clusters near the ventricles suggest a mitotic abnormality within periventricular germinal cells and the ensuing migration of daughter cells to the cortex. The resultant cortical abnormalities are of significance in helping explain some clinical aspects of autism, e.g., seizures, sensory hypersensitivity. It should be noted that although this study has focused on cortical abnormalities, disordered divisions of germinal cells within the rhombic lip may similarly account for brainstem malformations.

## Competing interests

The authors declare that they have no competing interests.

## Authors’ contributions

MFC designed the experiment, oversaw its implementation, and wrote the final manuscript. ASE-B, FK, AE, and AS, developed the algorithm used in measuring cortical width and the segmentation of the photomicrograph montages. SSK assisted in demarcating the gray white matter outlines and acquired the photomicrograph montages. BAD and AA-McN assisted in demarcating the gray white matter outlines of the coronal slides. AES implemented the algorithms to perform both the Boolean operation and the granulometry analysis, and performed the statistical analysis. All authors read and approved the final manuscript.
